# Supplementary material for: Evaluation of ddRADseq for reduced representation metagenome sequencing
Source: PeerJ. 2017 Sep 19;5:e3837. doi: 10.7717/peerj.3837 (PMC5609526; doi:10.7717/peerj.3837)
Supplement: Supplemental Information 1 [file peerj-05-3837-s001.docx]

**SUPPLEMENTARY INFORMATION**

***Adapter Hybridization***

Individual complementary single stranded oligos (Adapter oligos + Stub oligos) were hybridized to form a double-stranded annealed adapter, which contains a single stranded overhang that is complementary to the restriction enzyme cut-site overhang (e.g. a “sticky-end”), the associated barcode with different phaser, and a priming site for the Illumina adapter. Two sets of annealed adapters were hybridized that corresponded to the NlaIII and the HpyCh4IV restriction enzyme cut-sites. Eight i5 NlaIII adapters and 12 i7 HpyCH4IV adapters were generated (Table 1). The annealing reaction was carried out by mixing 10µM of Adapter oligo, 10µM of Stub oligo, 10X annealing buffer (100mM Tris-HCL [pH 7.5], 1M NaCl, 10mM EDTA and UltraPure water) and top-up to a final volume of 100µL with UltraPure water (Life Technologies). The annealing reaction was carried out at 96°C for 5 minutes, followed by a slow cooling down of the reaction tubes to room temperature (25°C). This was carried out using a thermocycler with ramp cool down of the tube with the rate of 0.1°C per second. Alternatively, this can be achieved by incubation in a heating block with heating turned off and let it cool down to the room temperature slowly. The annealed adapters were stored at -20°C until use with a molarity of around 10µM by assuming the annealing reaction was 100% efficient.

The annealed adapter oligos were used to create the ddRADseq libraries in this study. Although we designed many oligo pairs, only three pairs were used in the reported study. The combinatorial use of those annealed i5 NlaIII and i7 HpyCH4IV adapters in library preparation means up to 96 unique samples can be multiplexed in a single sequencing lane.

Table 1 The designed annealed adapter oligos for metagenomic ddRADseq.

| **Adapter Oligos** | **Phaser** | **Stub Oligos** | **Annealed Oligos** |  |
| --- | --- | --- | --- | --- |
|  |  |  |  |  |
| 1. i5 NlaIII ACTCTC | 0 | i5 NlaIII Phaser0 stub | *1.i5NlaIIIACTC* |  |
| 2. i5 NlaIII ATCCGG | 1 | i5 NlaIII Phaser1 stub | *2.i5NlaIIIATCC* |  |
| 3. i5 NlaIII GAGGAC | 2 | i5 NlaIII Phaser2 stub | *3.i5NlaIIIGAGG* |  |
| 4. i5 NlaIII ACCGGC | 3 | i5 NlaIII Phaser3 stub | *4.i5NlaIIIACCG* |  |
| 5. i5 NlaIII TGCCGT | 0 | i5 NlaIII Phaser0 stub | *5.i5NlaIIITGCC* |  |
| 6. i5 NlaIII AGGCTT | 1 | i5 NlaIII Phaser1 stub | *6.i5NlaIIIAGGC* |  |
| 7. i5 NlaIII ATAACC | 2 | i5 NlaIII Phaser2 stub | *7.i5NlaIIIATAA* |  |
| 8. i5 NlaIII GGAGGC | 3 | i5 NlaIII Phaser3 stub | *8.i5NlaIIIGGAG* |  |
|  | | | |  |
| **Adapter Oligos** | **Phaser** | **Stub Oligos** | **Annealed Oligos** |  |
|  |  |  |  |  |
| 1.i7 HpyCH GAGAGT | 0 | i7 HpyCH Phaser0 stub | *1.i7HpyGAGT* |  |
| 2.i7 HpyCH CCGGAT | 1 | i7 HpyCH Phaser1 stub | *2.i7HpyGGAT* |  |
| 3.i7 HpyCH GTCCTC | 0 | i7 HpyCH Phaser0 stub | *3.i7HpyCCTC* |  |
| 4.i7 HpyCH GCCGGT | 1 | i7 HpyCH Phaser1 stub | *4.i7HpyCGGT* |  |
| 5.i7 HpyCH ACGGCA | 0 | i7 HpyCH Phaser0 stub | *5.i7HpyGGCA* |  |
| 6.i7 HpyCH AAGCCT | 1 | i7 HpyCH Phaser1 stub | *6.i7HpyGCCT* |  |
| 7.i7 HpyCH GGTTAT | 0 | i7 HpyCH Phaser0 stub | *7.i7HpyTTAT* |  |
| 8.i7 HpyCH GCCTCC | 1 | i7 HpyCH Phaser1 stub | *8.i7HpyCTCC* |  |
| 9.i7 HpyCH AGGTCG | 0 | i7 HpyCH Phaser0 stub | *9.i7HpyGTCG* |  |
| 10.i7 HpyCH GACGAC | 1 | i7 HpyCH Phaser1 stub | *10.i7HpyCGAC* |  |
| 11.i7 HpyCH TTGATC | 0 | i7 HpyCH Phaser0 stub | *11.i7HpyGATC* |  |
| 12.i7 HpyCH CGCAAC | 1 | i7 HpyCH Phaser1 stub | *12.i7HpyCAAC* |  |

***Construction of ddRADseq libraries***

This ddRADseq protocol is a variation of the original Peterson et al 2012 protocol. The detail of the protocol is outlined as follow. Three healthy adult human stool samples were collected and DNA extracted using an UltraClean Microbial DNA isolation kit (MO BIO Laboratories) following the recommended protocol. The concentrations of DNA samples were examined using the Qubit HS DNA kit on a Qubit fluorimeter (Invitrogen) and diluted to a final concentration of 5ng/µL using UltraPure Water (Invitrogen). A total of 50ng of DNA from each sample was used for the restriction digest: 10µL of DNA (5ng/µL) was mixed with 10µL of restriction digest mix (1X NEB CutSmart buffer [New England Biolabs], 5U NlaIII [0.5µL of 10,000 U/mL, NEB], 5U HpyCh4IV [0.5µL of 10,000U/mL] and UltraPure Water). The digests were incubated at 37°C for 15 minutes and then heat inactivated at 65°C for 20 minutes. Annealed adapter ligation was performed at room temperature (25°C) for 15 minutes with 10U T4 DNA Ligase (New England Biolabs). The ligation mixture was made up with 1µL of the double digested DNA sample (~190nM), 7.6µM of NlaIII annealed adapter, 7.6µM of HpyCh4IV annealed adapter, 1X T4 Ligase buffer (New England Biolabs) and topped up to 20µL with UltraPure water. The mixture was heat deactivated by incubation at 65°C for 15 minutes using a water bath. This step can be performed using a PCR machine. Amplification of the adapter ligated DNA was carried out with the ddRADseq library amplification F5 and F7 primer oligo pair. An aliquot of 0.3ng of ligated DNA was used as input to the PCR reaction and mixed with 1µL of Illumina P7 adapter primer (1µM), 1µL of Illumina P5 adapter primer (1µM), 10µL of 2X KAPA HiFi Library Amplification Kit (KAPA Biosystems) and 6µL UltraPure water. Thermal cycling was carried out at 72°C for 3 minutes, 98°C for 30 seconds, followed by 20 cycles of 98°C for 15 seconds, 55°C for 30 seconds and 72°C for 30 seconds, followed by a final extension at 72°C for five minutes.

***Pooling and size selection of amplified ddRADseq libraries***

An equal volume of each ddRADseq library sample was taken and pooled together. Pooled material was then size selected using a double-sided clean-up with (0.5×/0.6×) SPRIselect magnetic bead from Beckman Coulter according to the manufacturer’s protocol. The resulted pooled DNA was then quantified with the Agilent 2100 Bioanalyzer using High Sensitivity DNA analysis kit (Aglient Technologies). ddRADseq libraries were pooled with unrelated samples and sequenced on an Illumina MiSeq with a 150 cycle V3 kit to generate paired-end 75nt reads. Shotgun metagenomic libraries were prepared from separate aliquots of sample DNA using the Illumina Nextera DNA kit. Sequencing of those samples was completed with half of a lane on the Illumina HiSeq 2500 platform using rapid PE250 mode.

***Full adapter oligo sequences***

*i5 NlaIII adapter oligo sequences:*

1.**i5 NlaIII ACTCTC**
AATGATACGGCGACCACCGAGATCTACACACTCTCNNNNNNACACTCTTTCCCTACACGACGCTCTTCCGATCTCATG

2.**i5 NlaIII ATCCGG**
AATGATACGGCGACCACCGAGATCTACACATCCGGNNNNNNACACTCTTTCCCTACACGACGCTCTTCCGATCTACATG

3.**i5 NlaIII GAGGAC**
AATGATACGGCGACCACCGAGATCTACACGAGGACNNNNNNACACTCTTTCCCTACACGACGCTCTTCCGATCTGTCATG

4.**i5 NlaIII ACCGGC**
AATGATACGGCGACCACCGAGATCTACACACCGGCNNNNNNACACTCTTTCCCTACACGACGCTCTTCCGATCTTGGCATG

5.**i5 NlaIII TGCCGT**
AATGATACGGCGACCACCGAGATCTACACTGCCGTNNNNNNACACTCTTTCCCTACACGACGCTCTTCCGATCTCATG

6.**i5 NlaIII AGGCTT**
AATGATACGGCGACCACCGAGATCTACACAGGCTTNNNNNNACACTCTTTCCCTACACGACGCTCTTCCGATCTACATG

7.**i5 NlaIII ATAACC**
AATGATACGGCGACCACCGAGATCTACACATAACCNNNNNNACACTCTTTCCCTACACGACGCTCTTCCGATCTGTCATG

8.**i5 NlaIII GGAGGC**
AATGATACGGCGACCACCGAGATCTACACGGAGGCNNNNNNACACTCTTTCCCTACACGACGCTCTTCCGATCTTGGCATG

*i5 NlaIII stub oligo sequences*

1.**i5 NlaIII Phaser0 stub** AGATCGGAAGAGCGTCGTGTAGGGAAAGAGTGT

2.**i5 NlaIII Phaser1 stub** TAGATCGGAAGAGCGTCGTGTAGGGAAAGAGTGT

3.**i5 NlaIII Phaser2 stub** ACAGATCGGAAGAGCGTCGTGTAGGGAAAGAGTGT

4.**i5 NlaIII Phaser3 stub** CCAAGATCGGAAGAGCGTCGTGTAGGGAAAGAGTGT

*i7 HpyCH4IV adapter oligo sequences:*

1.**i7 HpyCH ACTCTC**
CGAGATCGGAAGAGCACACGTCTGAACTCCAGTCACACTCTCNNNNNNATCTCGTATGCCGTCTTCTGCTTG

2.**i7 HpyCH ATCCGG**CGAAGATCGGAAGAGCACACGTCTGAACTCCAGTCACATCCGGNNNNNNATCTCGTATGCCGTCTTCTGCTTG

3.**i7 HpyCH GAGGAC**
CGAGATCGGAAGAGCACACGTCTGAACTCCAGTCACGAGGACNNNNNNATCTCGTATGCCGTCTTCTGCTTG

4.**i7 HpyCH ACCGGC**
CGAAGATCGGAAGAGCACACGTCTGAACTCCAGTCACACCGGCNNNNNNATCTCGTATGCCGTCTTCTGCTTG

5. **i7 HpyCH TGCCGT**
CGAGATCGGAAGAGCACACGTCTGAACTCCAGTCACTGCCGTNNNNNNATCTCGTATGCCGTCTTCTGCTTG

6. **i7 HpyCH AGGCTT**
CGAAGATCGGAAGAGCACACGTCTGAACTCCAGTCACAGGCTTNNNNNNATCTCGTATGCCGTCTTCTGCTTG

7. **i7 HpyCH ATAACC**
CGAGATCGGAAGAGCACACGTCTGAACTCCAGTCACATAACCNNNNNNATCTCGTATGCCGTCTTCTGCTTG

8. **i7 HpyCH GGAGGC**
CGAAGATCGGAAGAGCACACGTCTGAACTCCAGTCACGGAGGCNNNNNNATCTCGTATGCCGTCTTCTGCTTG

9. **i7 HpyCH CGACCT**
CGAGATCGGAAGAGCACACGTCTGAACTCCAGTCACCGACCTNNNNNNATCTCGTATGCCGTCTTCTGCTTG

10. **i7 HpyCH GTCGTC**
CGAAGATCGGAAGAGCACACGTCTGAACTCCAGTCACGTCGTCNNNNNNATCTCGTATGCCGTCTTCTGCTTG

11.**i7 HpyCH GATCAA**
CGAGATCGGAAGAGCACACGTCTGAACTCCAGTCACGATCAANNNNNNATCTCGTATGCCGTCTTCTGCTTG

12.**i7 HpyCH GTTGCG**
CGAAGATCGGAAGAGCACACGTCTGAACTCCAGTCACGTTGCGNNNNNNATCTCGTATGCCGTCTTCTGCTTG

*i7 HpyCH4IV stub oligo sequences:*

1.**i7 HpyCH Phaser0 stub**: GTGACTGGAGTTCAGACGTGTGCTCTTCCGATCT

2.**i7 HpyCH Phaser1 stub**: GTGACTGGAGTTCAGACGTGTGCTCTTCCGATCTT

*ddRADseq library amplification oligo sequences*

**P5 primer oligo**: AATGATACGGCGACCACCGA

**P7 primer oligo**: CAAGCAGAAGACGGCATACGA
